# Genome Editing for CNS Disorders

**DOI:** 10.3389/fnins.2020.579062

**Published:** 2020-10-22

**Authors:** Fábio Duarte, Nicole Déglon

**Affiliations:** ^1^Laboratory of Neurotherapies and NeuroModulation, Department of Clinical Neurosciences, Lausanne University Hospital and Lausanne University, Lausanne, Switzerland; ^2^Laboratory of Neurotherapies and NeuroModulation, Neuroscience Research Center, Lausanne University Hospital and Lausanne University, Lausanne, Switzerland

**Keywords:** CNS, **g**enome editing, ZFs, TALEs, CRISPR/Cas

## Abstract

Central nervous system (CNS) disorders have a social and economic burden on modern societies, and the development of effective therapies is urgently required. Gene editing may prevent or cure a disease by inducing genetic changes at endogenous loci. Genome editing includes not only the insertion, deletion or replacement of nucleotides, but also the modulation of gene expression and epigenetic editing. Emerging technologies based on ZFs, TALEs, and CRISPR/Cas systems have extended the boundaries of genome manipulation and promoted genome editing approaches to the level of promising strategies for counteracting genetic diseases. The parallel development of efficient delivery systems has also increased our access to the CNS. In this review, we describe the various tools available for genome editing and summarize *in vivo* preclinical studies of CNS genome editing, whilst considering current limitations and alternative approaches to overcome some bottlenecks.

## Introduction

Neurological disorders are the principal cause of disability and the second leading cause of death worldwide (Feigin et al., 2019). Central nervous system (CNS) diseases include diverse infections (meningitis and encephalitis), vascular disorders (stroke and other hemorrhages), structural (brain or spinal injury), functional (epilepsy and migraines) and neurodegenerative (Alzheimer’s and Parkinson’s disease) conditions. With steady increases in the size and age of the world population, the prevalence of these diseases is likely to increase, and they have thus become a priority area of research. The age-standardized frequencies of neurological diseases have declined, but the number of people affected worldwide has continued to increase. The continual aging of the population is, thus, outstripping our ability to counteract these disorders (Feigin et al., 2019).

The development of therapeutic strategies for CNS disorders is challenging, given the considerable diversity of cells involved, the extreme complexity of the neural circuits and associated functions, poor tissue regeneration and our incomplete understanding of the underlying pathological processes. Pharmacological efficacy depends on our ability to take all of these factors into account. For some disorders, such as traumatic and neurodegenerative conditions, the timing of treatment may also be important, with therapeutic success decreasing as neurodegeneration progresses. Moreover, the blood–brain barrier (BBB) limits the diffusion of most molecules delivered by conventional methods. Consequently, the doses of drugs delivered systemically often have to be increased to ensure that therapeutic concentrations are reached in the CNS, which may lead to toxicity.

As a result of these challenges, approval rates are much lower for CNS-targeting drugs than for drugs targeting other parts of the body (Kesselheim et al., 2015; Gribkoff and Kaczmarek, 2017). This has led to a revision of CNS drug development guidelines and to the implementation of innovative and efficient therapeutic models. One particular treatment strategy, gene therapy, has progressed remarkably over the last 15 years. It involves the introduction of recombinant nucleic acids into the patient’s cells, to fight or prevent a disease (Klug et al., 2012). Two different therapeutic approaches can be used: *ex vivo* and *in vivo*. *Ex vivo* gene therapy entails: (1) the collection of cells from the patient, (2) the culture and modification of these cells *in vitro* and (3) the transplantation of the modified cells back into the recipient. The *in vivo* approach involves modification of cells directly in the individual. One of the major advantages of gene therapy is that it can be used to modify most biological pathways through the targeting of the underlying genes. It can halt or reverse disease progression by targeting the underlying pathogenic processes, whereas conventional medicine often focuses on symptom relief. In addition, stable transgene expression or permanent genome modification may make it possible to treat disorders in a single administration.

The most straightforward application of gene therapy is the treatment of monogenic disorders. A classical approach to the treatment of diseases caused by loss-of-function (LOF) mutations is based on the replacement of the defective gene with the wild-type (WT) cDNA. The treatment of familial lipoprotein lipase deficiency (LPLD) with Glybera (a rAAV1 encoding the lipoprotein lipase variant LPLS447X; Ylä-Herttuala, 2012), a rare form of inherited blindness with Luxturna (a rAAV2 encoding a normal copy of the retinal pigment epithelium-specific 65 kDa protein; Russell et al., 2017), and spinal muscular atrophy (SMA) with Zolgensma (a rAAV9 encoding the survival motor neuron 1 protein; Hoy, 2019) provide examples of approved products. The treatment of autosomal dominant disorders caused by gain-of-function (GOF) mutations generally involves decreasing the levels of mutant mRNA by RNA interference (RNAi) or with antisense oligonucleotides (ASO). An example is provided by mipomersen, an ASO targeting the apolipoprotein B mRNA, which can be used to treat homozygous familial hypercholesterolemia (Hair et al., 2013). Gene therapy products have been also developed to combat genes in which the pathological mutation alters transcript splicing. Examples include eteplirsen for Duchenne muscular dystrophy (DMD) (Syed, 2016) and nusinersen (Spinraza^®^) for SMA (Hoy, 2017). These products deliver nucleic acids targeting the mutant primary transcripts, and modify the splicing of these transcripts into non-pathogenic isoforms. Finally, gene-based therapeutic approaches have also been successfully used for the treatment of polygenic diseases, such as cancer and infectious diseases (Shahryari et al., 2019). In such cases, the strategies developed target one of the identified pathogenic genes (LOF or GOF) or deliver transgenes encoding factors with protective functions.

Gene replacement approaches have been successfully applied to some disorders, but (1) the size of the transgene may be limited by the delivery system, (2) this approach is usually restricted to the expression of a single gene isoform and (3) the lack of a transgene chromatin signature often results in non-physiological levels of expression (Khabou et al., 2018). Conversely, gene silencing with RNAi/ASO (1) does not completely knockout the pathological gene, and a total knockout may be essential for highly damaging genes, (2) its therapeutic efficacy depend on the turnover of the targeted transcript and (3) may require continuous drug administration to maintain the therapeutic benefit (Sledz and Williams, 2005).

Genome editing has emerged as a complementary gene therapy strategy. It operates at native DNA loci, and can be used for the complete inactivation of a toxic gene, gene repair or regulation of an endogenous gene (Doudna, 2020). Genome editing tools have been available for 30 years, but their limited efficacy, complex production and the lack of efficient delivery vehicles have delayed their clinical application. Over the last decade, more sophisticated and precise editing tools have rendered genome engineering not only promising for gene-based therapeutic approaches, but also useful as a technique for basic biology, genetic diagnosis and drug discovery purposes (Doudna, 2020; Li et al., 2020; Sandoval et al., 2020; Wertz et al., 2020). Indeed, therapeutic genome editing is no longer a concept for the distant future, and several *ex vivo* and *in vivo* therapeutic approaches are currently undergoing clinical testing for the treatment of various diseases (Schacker and Seimetz, 2019; Li et al., 2020). In this review, we describe the various genome editing tools available and summarize some of the preclinical studies of *in vivo* CNS genome editing published to date, while discussing current limitations and alternative approaches to overcome some of the bottlenecks.

## DNA-Binding Platforms

Editing platforms have two key features essential for the specific and efficient modification of target sequences within the genome: (1) a DNA-binding domain recognizing a unique target sequence and (2) an effector element for inducing precise genetic/epigenetic modifications. The genome editing tools currently available are based on three major DNA-binding platforms: zinc fingers (ZFs), transcription-activator like effectors (TALEs), and clustered regularly interspaced short palindromic repeats (CRISPR/Cas).

Zinc fingers are eukaryotic DNA-binding domains consisting of two anti-parallel β-sheets and one α-helix, the residue composition of which specifies binding to particular triplets (Miller et al., 1985; Pavletich and Pabo, 1991). Merging six ZFs, is sufficient to create larger DNA-recognition domains targeting unique sequences (18 base pairs) in eukaryotic genomes (Urnov et al., 2010). The construction of extensive libraries of ZFs has made it possible to engineer zinc finger proteins (ZFPs) targeting almost any sequence desired.

Transcription-activator like effectors were first discovered in *Xanthomonas*, a plant-pathogenic bacterium (Bonas et al., 1989; Boch and Bonas, 2010). These proteins bind the DNA via a central region containing an array of 33- to 35-amino acid motifs. The amino-acid sequences of arrays are similar except for two positions, conferring nucleotide-binding specificity. Unlike ZFs, in which each domain recognizes a specific trinucleotide, each TALE array recognizes a single nucleotide.

CRISPR/Cas are the most recently developed tools for genome engineering. They are based on an RNA-guided nuclease, the DNA-binding properties of which are easily modulated by a short RNA sequence (Fineran and Charpentier, 2012; Wiedenheft et al., 2012). They are involved in bacterial adaptable immunity and can be grouped into two main classes according to the complexity of the nuclease effector (Makarova et al., 2015, 2020). Class 1 systems (types I, III, and IV) involve a large complex of several effector proteins, whereas class 2 systems (types II, V, and VI) use a single Cas protein to mediate the recognition and cleavage of foreign nucleic acids. Class 2 systems are the most widely used for genome editing, because of their simple structure. Type II and type V CRISPR/Cas ribonucleoprotein complexes recognize specific DNA sequences through RNA-DNA base pairing. Cas binding and interference are determined by the spacer sequence (∼20 bp) of the single guide RNA (sgRNA), and the protospacer adjacent motif (PAM) on the target DNA. The spacer is complementary to the target sequence and the PAM is a short DNA motif immediately adjacent to the target region. Cas9 (type II) and Cas12a (type V) have been extensively explored for genome editing (Jinek et al., 2012; Cong et al., 2013; Mali et al., 2013; Zetsche et al., 2015). Cas9 requires a 3′ PAM to the target sequence, whereas Cas12a recognizes a 5′ PAM on the non-targeted strand. Cas9 induces PAM-proximal blunt double-strand breaks (DSBs) and Cas12a creates PAM-distal staggered DSBs. PAM requirements make it impossible for a single CRISPR/Cas system to target all genomic sequences, but the use of different Cas9 and Cas12a orthologs with different PAM specifications has greatly expanded targeting capabilities (Cebrian-Serrano and Davies, 2017). In addition, Cas9 proteins have been engineered to accept different and less restrictive PAMs, although sometimes compromising the specificity (Hu et al., 2018; Walton et al., 2020). By contrast, other groups have restricted Cas9 binding parameters to increase specificity, which however reduce the editing efficiency (Kleinstiver et al., 2016; Kocak et al., 2019). The major advantage of CRISPR/Cas-based tools over ZFs and TALEs for genome editing is the ease of engineering of the DNA-binding domain to recognize unique sequences. The DNA-binding specificity of ZFs and TALEs is dependent on protein-DNA interactions and the targeting of particular sequences therefore requires protein design. The genome-targeting specificity of CRISPR/Cas is provided by the sgRNAs, which are simpler and less expensive to design.

## Fusing DNA-Binding Domains to Effector Domains: Genome Editing Approaches

Genome editing can be grouped into four approaches, depending on the effector domains used (Table 1). The DNA sequence can be permanently altered by gene editing or base editing, whereas a transient or stable modification of DNA function/expression can be achieved with gene regulation or epigenetic editing.

**TABLE 1:** Comparison of the different genome editing approaches.

Editing approaches	Advantages	Disadvantages
Gene editing	Efficient Permanent All possible modifications: insertion, deletion and substitution	Off-target cleavage Chromosomal instability Target sequence restriction (PAM for CRISPR; 5’-T for TALENs) NHEJ is heterogeneous HDR is inefficient (especially in post-mitotic cells)
Base editing	Permanent No need to induce DSBs Few or no indels	Off-target at both DNA and RNA level Bystander base editing Target sequence restriction (PAM) Efficiency is low Only substitutions are possible
Transcriptional regulation	Physiological expression level Low off-target effects Cell reprogramming	Efficacy depends on the level of gene expression Large genomic areas can be affected Most modifications are not permanent
Epigenetic editing	Long-term modification Cell reprogramming	Lack of information on epigenetic marks for some targeted genes May affect large genomic regions Simultaneous modification of several epigenetic marks may be necessary

### Gene Editing

**FIGURE 1:**
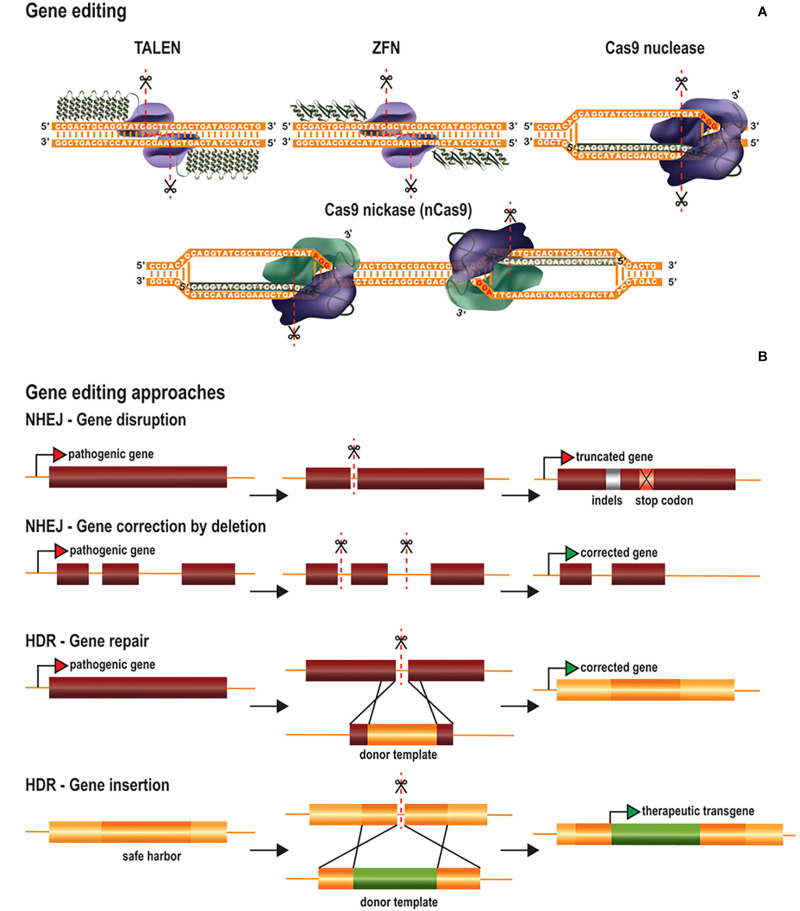
Gene editing tools and therapeutic approaches. **(A)** Gene editing tools are based on TALEs, ZFs and CRISPR/Cas platforms. Site-specific TALENs and ZFNs consist of two modules of TALEs and ZFs fused to the *Fok*I nuclease. Both modules recognize adjacent sequences in opposite strands to promote the dimerization of *Fok*I and sequence cleavage in a staggered fashion. In contrast, CRISPR/Cas systems hold intrinsic nuclease activity. Cas nucleases or Cas nickases are explored to produce either DSBs or SSBs in the targeted sequence, respectively. Alternatively, paired nickases targeting adjacent sequences in opposite strands generate staggered DSBs. **(B)** Gene editing therapeutic approaches rely on the intrinsic DNA repair mechanisms NHEJ and HDR after generation of DSBs. Gene disruption by NHEJ involves the introduction of indels after generation of DSBs at the coding region of a pathogenic gene, resulting in the formation of a premature stop codon. Gene correction by NHEJ implicates the targeting of the non-coding region of a pathogenic gene. It includes the removal of deleterious exons by the simultaneous cleavage in both upstream and downstream intronic regions and/or disruption of splicing regulation sites. Both gene repair and gene insertion by HDR involve the use of donor templates containing intended sequences flanked by homology arms. In the first case, the template is targeted to the pathogenic gene and contains the corrected sequence allowing gene restoration. In contrast, gene insertion by HDR targets safe harbor locations in the genome to introduce therapeutic transgene expression cassettes.

The effector domain of gene editing platforms is a nuclease that induces DSBs at the target DNA sequence (Doudna, 2020; Li et al., 2020). Cas proteins possess intrinsic nuclease activity, whereas ZF nucleases (ZFNs) and TALE nucleases (TALENs) have been engineered by fusing the catalytic domain of the *Fok*I nuclease to ZFs and TALEs, respectively (Figure 1A). *Fok*I is a bipartite endonuclease that must dimerize to cleave the target sequence (Vanamee et al., 2001). ZFNs and TALENs therefore have two fused *Fok*I domains binding opposite strands of adjacent sequences in reverse orientations, to promote *Fok*I dimerization and genome restriction (Figure 1A). Spatial orientation and module spacing requirements decrease the probability of off-target cutting events. Site-specific DNA cleavage activates cellular DNA repair pathways, which then delete, insert or replace nucleotide sequences (Yeh et al., 2019). The two main DNA repair pathways for DSBs are the non-homologous end-joining (NHEJ) and homology-directed repair (HDR) pathways. NHEJ is error-prone, often introducing small insertions or deletions (indels), whereas HDR uses homologous sequences as a template, to ensure the correct repair of damaged DNA (Figure 1B). The NHEJ pathway is frequently used to inactivate toxic genes (Figure 1B). The introduction of indels at the 5′ end of the target gene results in frameshift mutations, generating premature stop codons. Other applications include the disruption of aberrant splicing sites or the deletion of large fragments of DNA through the creation of two DSBs in the same chromosome (Figure 1B). By contrast, the accuracy of the HDR pathway allows precise nucleotide insertions, deletions or substitutions at the target site (Figure 1B). This is achieved by using double- or single-stranded DNA templates containing the intended modification, flanked by homologous sequences. HDR can, thus, be used to correct both GOF and LOF mutations, for gene repair. HDR can also be exploited as an alternative approach to classical gene replacement, to improve control over the copy number of the gene of interest and to prevent insertional mutagenesis due to the random integration of viral vectors. HDR-mediated gene replacement involves the site-specific insertion of full transgenes (cDNA) at “safe harbor” locations, defined as sites within the genome at which the addition of sequences does not interfere with the neighboring genes and results in safe robust transgene expression (Figure 1B).

HDR-mediated gene editing is a promising approach for therapeutic applications, but it is generally less efficient than NHEJ and mostly restricted to the G2 and S phases of the cell cycle (Yeh et al., 2019). This imposes additional challenges for the application of HDR-based editing to post-mitotic cells and, therefore, to CNS disorders. Nishiyama and colleagues reported a high efficiency of HDR in the mouse brain (Nishiyama et al., 2017), but most groups have struggled to achieve such success with this approach. Several groups have proposed NHEJ-like strategies to overcome this limitation through precise gene editing in non-replicative cells by microhomology-mediated end-joining (MMEJ) (Yao et al., 2017b), homology-independent targeted integration (HITI) (Suzuki et al., 2016), and microhomology-dependent targeted integration (MITI) (Li et al., 2019). Other groups have explored HDR-like mechanisms, such as homology-mediated end joining (HMEJ) (Yao et al., 2017a) and single homology arm donor-mediated intron-targeting integration (SATI) (Suzuki et al., 2019). These techniques have yielded significantly higher rates of gene insertion in post-mitotic cells, although the mechanisms involved are not fully understood. Other groups have suggested approaches in which HDR repair is promoted by fusing the Cas9 nuclease to factors involved in the regulation of NHEJ/HDR pathways. For instance, p53-binding protein 1 (53BP1), which plays a major role in balancing NHEJ/HDR ratio, promotes DSB repair via the NHEJ pathway by preventing the DNA end resection required for HDR (Bunting et al., 2010). Cas9 fused to a dominant-negative 53BP1 enhances HDR and inhibits NHEJ in a target-specific manner, without modifying cellular DNA repair mechanisms overall (Jayavaradhan et al., 2019). Efforts have also been made to improve HDR by fusing Cas9 to RecA (RAD51 in eukaryotes), which plays a key role in homologous recombination (Cai et al., 2019; Kurihara et al., 2020), or by altering the conformational checkpoints for Cas9 binding to DNA (Kato-Inui et al., 2018).

No product for therapeutic gene editing has yet been approved, but the first clinical trials based on this technology have demonstrated the safety of this approach (Schacker and Seimetz, 2019). However, as gene editing permanently modifies the DNA, several biosafety concerns have been raised concerning the induction of off-target DSBs and increases in genomic instability (Mills et al., 2003). Unlike DSBs, DNA single-strand breaks (SSBs) are common events under physiological conditions, and are less harmful than DSBs (Caldecott, 2008). Nickases were developed by mutating one of the catalytic sites of Cas9 (nCas9), such that only one strand of the DNA is cut (Doudna, 2020) (Figure 1A). Paired nickases targeting nearby sequences on opposing strands can create specific DSBs, while decreasing the chances of producing off-target DSBs (Dabrowska et al., 2018; Ge and Hunter, 2019). The use of SSBs and ssDNA repair templates to insert specific sequences has been explored as an alternative to DSB-mediated HDR (Rees et al., 2019). Nickase variants have improved the HDR:indel ratio, but, overall, this approach remains less efficient than DSB-mediated recombination.

## Base Editing

**FIGURE 2:**
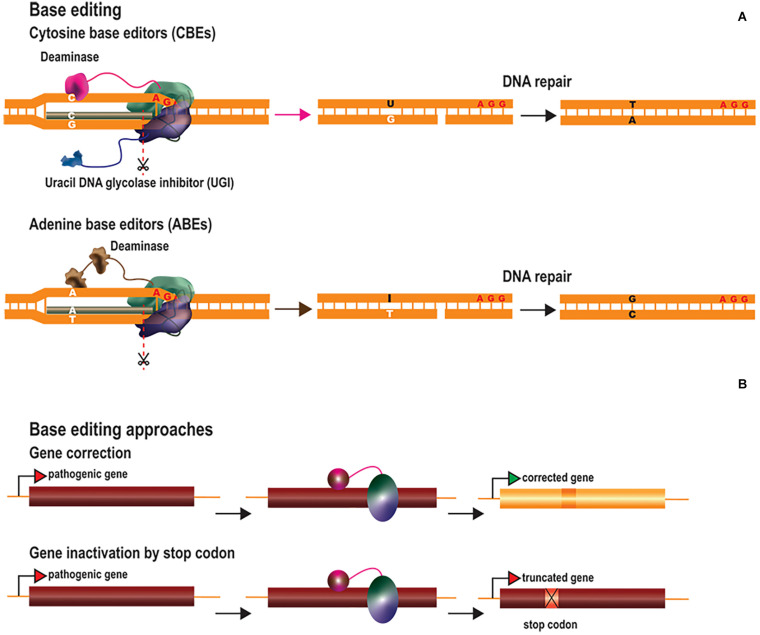
Base editing tools and therapeutic approaches. **(A)** Base editors consist of Cas nickases fused to cytosine (CBEs) or adenine ssDNA deaminases (ABEs). CBEs are fused to either AID or APOBEC1 (pink), which convert C into U, whereas ABEs are fused to an evolved TadA (TadA*) followed by a wild-type TadA fusion (brown), which convert A into I. The consequent G:U and T:I mismatches are then corrected by the cellular DNA repair mechanisms. To favor the correction of the non-edited nucleotides by the DNA mismatch repair machinery, the nickase introduces a “nick” in the unedited strand. The correction of the non-edited strand results in a final conversion of C:G into T:A base pairs and A:T into G:C base pairs by CBEs and ABEs, respectively. CBEs are usually fused to the UGI to prevent the rapid removal of uracil by BER (blue). **(B)** Base editing therapeutic approaches include the repair of pathogenic genes by correcting point mutations or the inactivation of toxic genes by generating a premature stop codon.

DNA base editing can be used to modify single nucleotides without the need to introduce DSBs, reducing the risk of creating off-target indels (Rees and Liu, 2018; Molla and Yang, 2019). Base editing could potentially be used to correct pathogenic point mutations, the most common type of human genetic disorders (Landrum et al., 2016). DNA base editors have been generated by fusing catalysis-deficient Cas9 (dCas9) or nCas9 to deaminase enzymes, which convert specific nucleotides (Komor et al., 2016; Nishida et al., 2016; Gaudelli et al., 2017) (Figure 2A). These tools make use of the sgRNA/Cas-mediated R-loop structure to target the transient ssDNA with cytosine or adenosine deaminases. Cytosine base editors (CBEs) convert cytosine into uracil (C→U), which has similar base-pairing properties to thymine (T). The U is then converted to T via DNA repair mechanisms based on base excision repair (BER) or mismatch repair (MMR), resulting in the conversion of C⋅G into T⋅A base pairs. The first generation of CBEs (BE1) was developed by fusing dCas9 to the apolipoprotein B mRNA-editing enzyme, catalytic polypeptide 1 (APOBEC1) (Komor et al., 2016). This tool converted cytosine nucleotides in the test tube, but not in eukaryotic cells. The authors rapidly realized that the poor cytosine conversion in cells might be due to intrinsic U:G mismatch repair mechanisms. Uracil is one of the most common non-canonical bases in DNA and its removal by DNA repair mechanisms is important, to prevent mutagenesis. Uracil removal is initiated by uracil DNA glycolase (UDG), which excises the uracil and triggers the conversion of U:G into C:G base pairs by BER. Consequently, the second generation of CBEs (BE2) were fused to a uracil DNA glycosylase inhibitor (UGI), to prevent uracil base excision repair, considerably improving cytosine editing. Attempts were then made, in the third generation of CBEs (BE3) to favor the incorporation of the modified nucleotide through the use of nCas9 rather than dCas9, to induce a “nick” in the unedited strand, thereby favoring the correction of the non-edited nucleotides by the DNA mismatch repair machinery. This resulted in higher cytosine conversion efficiencies, but also increased the frequency of indel events. Indel formation in this context probably results from the creation of two adjacent DNA nicks on opposite strands (by nCas9 on the unedited strand and by BER enzymes on the edited strand), leading to the generation and NHEJ-mediated processing of transient DSBs. For this reason, a fourth generation of CBEs was generated by fusing nCas9 to two UGIs (BE4) and/or to the bacteriophage Mu-derived Gam (BE4-GAM), which binds to DSBs and protects them from degradation (Komor et al., 2017). The BE4 editors underwent further improvement, based on the modification of nuclear localization signals, codon optimization and deaminase reconstruction (BE4max) (Koblan et al., 2018). In parallel, CBEs were generated with an ortholog of activation-induced cytidine deaminase A (AID) from sea lamprey (PmCDA1) rather than APOBEC1 (Nishida et al., 2016). CDA1-nCas9-UGI had editing rates similar to those of APOBEC1-nCas9-UGI, but achieved through periodic decreases in incubation temperature to 25°C, the optimal temperature for PmCDA1. An extended toolbox of DNA CBEs is now available. These editors differ in terms of their Cas proteins (Cas9 or Cas12a), nuclease activity (dCas or nCas), cytosine deaminase (APOBEC1 or CDA1), number of UGIs, nuclear localization signals and the linker sizes between domains (Rees and Liu, 2018; Molla and Yang, 2019).

Adenine base editors (ABEs) transform adenine into ionosine (A→I), which is then converted to guanine (G), resulting in the conversion of A⋅T into G⋅C base pairs (Figure 2A). ABEs were generated based on the tRNA adenine deaminase (TadA) of *Escherichia coli* (Gaudelli et al., 2017). After several rounds of development, it was established that the fusion of nCas9 to an evolved TadA (TadA^∗^) followed by a wild-type TadA resulted in the most efficient ABE (ABE 7.10). The DNA repair mechanisms for removing ionosine from the DNA are less efficient than those for removing uracil, and these tools were, therefore, able to induce high levels of adenine conversion without the need for ionosine repair inhibitors. Consequently, indel events were much less frequent (barely detectable) than in untreated samples. As for BE4max, the efficiency of ABE7.10 was also increased by the development of ABEmax (Koblan et al., 2018). The TadA^∗^-TadA effector domain was fused to several Cas9 variants, recognizing different PAMs, to increase the breadth of targeting possible for ABEs (Rees and Liu, 2018; Molla and Yang, 2019).

Base editing is dependent on DNA mismatch repair rather than homologous recombination. It therefore constitutes an alternative approach to HDR-mediated gene editing for correcting point mutations in post-mitotic cells. CBEs and ABEs have been used to correct both LOF and GOF pathogenic point mutations implicated in various diseases (Komor et al., 2016; Gaudelli et al., 2017; Liang et al., 2017; Koblan et al., 2018; Zeng et al., 2018) (Figure 2B). *In vivo* base editing applications have been described for hypercholesterolemia (Chadwick et al., 2017; Rossidis et al., 2018), hearing loss (Yeh et al., 2018, 2020), hereditary tyrosinemia type 1 (Rossidis et al., 2018), phenylketonuria (Villiger et al., 2018), DMD (Ryu et al., 2018) and amyotrophic lateral sclerosis (ALS) (Lim et al., 2020). Another therapeutic strategy involves the generation of a premature stop codon for gene inactivation, as an alternative to NHEJ-mediated gene editing (Billon et al., 2017; Kuscu et al., 2017) (Figure 2B).

Base editing is a promising therapeutic strategy, but it is subject to limitations in terms of the purity of the edited products, bystander base editing and distal off-target activity. Product purity is defined as the ratio of intended to unintended editing events at the targeted site. Uracil is more prone to repair by base excision repair mechanisms, so product purity is lower for CBEs than for ABEs. This translates into a higher rate of C to non-T nucleotide conversion and indel events than of A to non-G conversions and indels generated by ABEs. Bystander base editing also lowers product purity by modifying base pairs adjacent to the targeted nucleotide. Bystander editing of adjacent Cs or As can be counteracted by employing base editors with narrow editing windows, although some such editors are less efficient. For distal off-target editing, CBEs have been shown to generate more off-target mutations than ABEs (Lee H.K. et al., 2018; Zuo et al., 2019). Base editors have been shown to induce unintended modifications in both DNA and RNA. Indeed, a recent report demonstrated substantial levels of off-target editing in RNA, for both CBEs and ABEs (Zhou et al., 2019). Distal off-target editing may result from non-specific Cas protein binding to DNA and RNA or random contacts between the deaminase domains and RNA or ssDNA during DNA replication and transcription (Rees and Liu, 2018; Molla and Yang, 2019). Cas-dependent off-target editing has been reduced by the use of high-fidelity Cas variants, and other types of off-target editing can be limited by altering the intrinsic DNA and RNA affinity of deaminase domains.

### Genome Regulation

**FIGURE 3:**
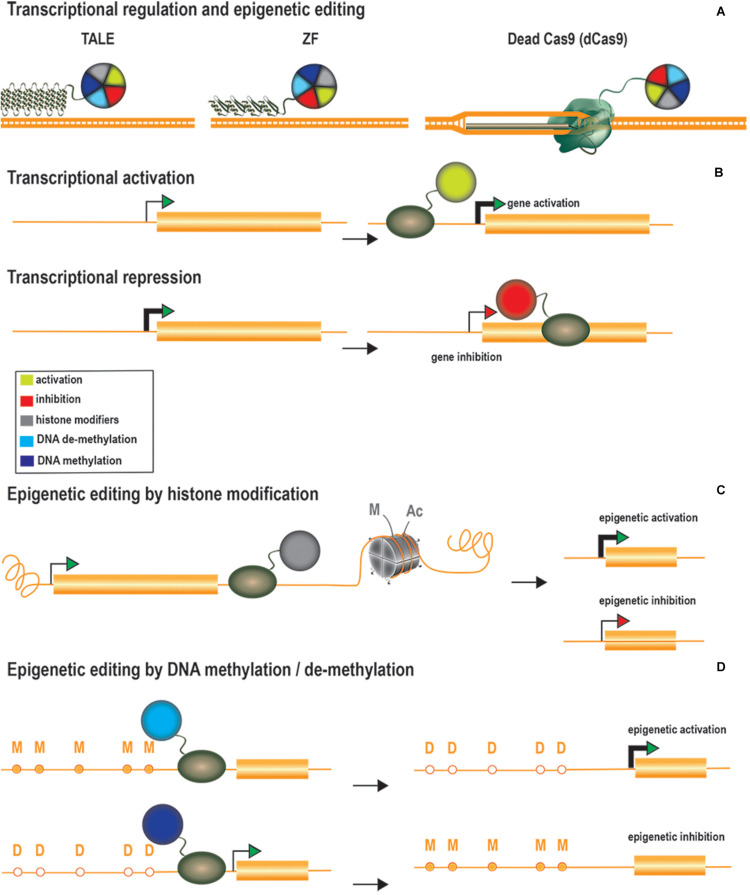
Transcriptional regulators, epigenetic modifiers, and therapeutic approaches. **(A)** Gene expression regulation tools are generated by fusing TALEs, ZFs or dCas proteins to scaffold transcriptional modulators or to epigenetic modifiers **(B)** Therapeutic approaches by transcriptional regulation. Transcriptional activation or repression is explored to upregulate therapeutic genes or to downregulate deleterious genes, respectively. Transcriptional activators are targeted at the promoter region whereas transcriptional repressors are usually targeted downstream to the transcription starting site to further block the RNA polymerase activity. **(C)** Therapeutic approaches through histone modification. Histone (de)acetylases and (de)methylases are the most common employed enzymes to modify histone marks and the epigenetic activation or inhibition effect of such modifications is frequently context-specific. **(D)** Therapeutic approaches by editing the DNA methylation state. Epigenetic editors based on DNA demethylases are used to activate gene expression whereas the ones based on DNA methylases result in gene expression inhibition.

Genome regulation offers additional therapeutic options through the modulation of gene expression at native loci. Gene expression is regulated by multiple factors, including both *cis* and *trans* elements, ultimately leading to the recruitment of RNA polymerases to promoter regions. Genome expression is also regulated by epigenetic marks, which determine chromatin accessibility state and comprise multiple elements, including the three-dimensional architecture of the DNA and histone or DNA modifications (Holtzman and Gersbach, 2018). An extensive list of possible histone modifications, including acetylation, methylation and phosphorylation, has been described, and all these processes can be altered to modulate gene expression (Holtzman and Gersbach, 2018). Epigenetic modifications, particularly for histone tails and DNA methylation status, have provided insight into the role of such changes in gene regulation and their contribution to disease. For instance, cytosine methylation (5C-methylcytosine) at CpG dinucleotides is usually enriched in silenced promoters (Weber et al., 2007; Kundaje et al., 2015) and has been implicated in genomic imprinting (Laan et al., 1999), whereas H3K9 acetylation is associated with active promoters (Ernst et al., 2011). For the alteration or restoration of gene expression profiles, ZFs, TALEs, and dCas proteins have been fused to scaffold transcriptional modulators or epigenetic modifiers (Figure 3A). Genome regulation strategies can be used to upregulate or repress gene expression by two different approaches: (1) transcriptional modulation through the recruitment of transcription factors and chromatin remodelers and (2) epigenome editing through the direct modification of epigenetic marks.

Transcriptional activation has been achieved through the tethering of ZFs, TALEs, and dCas9 to several copies of herpes simplex virus protein 16 (VP16), the transactivating domain of the NF-kB p65 subunit (p65), heat shock factor 1 (HSF1) and Epstein–Barr virus R transactivator (RTA) (Figure 3B). The targeting of multiple copies of transactivating domains to promoter regions was rapidly shown to have a synergistic activation effect. This led to the development of dCas-based second-generation activators, which can target multiple transactivating domains to a single locus. Chavez and coworkers evaluated the potency of several dCas9 activators in different cell lines and showed that the synergistic activator mediator (SAM) (Konermann et al., 2015), SUperNova Tagging (SunTag) (Tanenbaum et al., 2014) and the tripartite VP64-p65-RTA (VPR) (Chavez et al., 2015) systems were the most efficient at inducing gene activation (Chavez et al., 2016). These systems have been adapted to activate genes *in vivo* (Chew et al., 2016; Liao et al., 2017; Moreno et al., 2018; Zhou et al., 2018; Breinig et al., 2019; Savell et al., 2019; Zhan et al., 2019). Gene expression profiles can also be altered to reprogram cells to differentiate into particular cell types. Liao and coworkers reprogrammed hepatic cells into pancreatic-like beta cells, by activating the Pdx1 (Liao et al., 2017). They also improved DMD symptoms by activating the Utrn gene. Savell and coworkers demonstrated robust Fosb activation in several regions of the brain *in vivo* (Savell et al., 2019). Similarly, Zhou et al. demonstrated the *in vivo* genetic reprogramming of neurons in mouse brain by simultaneously activating the expression of Ascl1, Neurog2, and Neurod1 (Zhou et al., 2018). Breinig and coworkers recently altered the sgRNA length of a Cas12a-VPR variant to induce either gene activation or knockout *in vivo* (Breinig et al., 2019). This work has added an additional degree of complexity to these systems, allowing not only the targeting of multiple genes, but also a larger range of modifications. Artificial transcriptional repressors have also been generated by fusing the Kruppel-associated box protein (KRAB) domain to the DNA-binding platforms (Bailus et al., 2016; Zeitler et al., 2019) (Figure 3B). KRAB is a scaffold protein involved in recruiting KAP1/TIF1β corepressor complexes, which in turn recruit DNA methylases or histone modifier factors (Kim et al., 1996; Ying et al., 2015). The effects of KRAB on gene repression can be permanent or reversible, depending on developmental stage (Ying et al., 2015).

Unlike the recruitment of activating or repressing complexes/factors, epigenetic editing can modify epigenetic marks by targeting specific enzymes. Epigenetic activation has been achieved through site-specific DNA methylation by the DNA demethylase 10–11 translocation methylcytosine dioxygenase 1 (TET1) (Maeder et al., 2013; Choudhury et al., 2016a; Liu et al., 2016; Xu et al., 2016) or with enzymes promoting activating histone signatures, such as the histone acetylase core subunit p300 (Hilton et al., 2015) and histone methyltransferases (Cano-Rodriguez et al., 2016) (Figures 3C,D). For instance, Liu and coworkers described the *in vivo* demethylation of a methylation-sensitive Snrpn-GFP cassette in transgenic mice (Liu et al., 2016). Heterozygous mice carrying a paternal copy of the transgene do not express GFP, due to the methylated status of this copy of the gene. The authors reported the targeted active demethylation of the transgenic cassette and a 70% activation of GFP expression after lentiviral injections of dCas9-Tet1 into the brain. Rather than active DNA demethylation, Hilton et al. demonstrated that the fusion of p300 to the three DNA-binding platforms activated the expression of multiple endogenous genes (*ilrn1, oct4*, and *myod1*) through histone acetylation (Hilton et al., 2015). Finally, epigenetic repression has been achieved through direct DNA methylation (Bernstein et al., 2015), histone deacetylation (Kwon et al., 2017), and histone demethylation (Kearns et al., 2015) (Figures 3C,D).

## Genome Editing for CNS Disorders

### Alzheimer’s Disease

Alzheimer’s disease (AD) is the main cause of dementia, affecting millions of people worldwide (Winblad et al., 2016; Dos Santos Picanco et al., 2018). One of the hallmarks of AD is the presence of scattered extracellular senile plaques, due to the accumulation of amyloid-β (Aβ) in the brain. Aβ is a secondary metabolite generated by the processing of amyloid precursor protein (APP) by β-secretase 1 (BACE1). Alternatively, APP may be processed via a non-amyloidogenic pathway involving α-secretases, leading to the generation of neuroprotective products (Richter et al., 2018). In a study of the treatment of a familial form of AD caused by the Swedish mutation of APP (APPsw), CRISPR-mediated NHEJ was used to inactivate the mutant APP (György et al., 2018). This can be achieved by designing sgRNAs targeting single nucleotide polymorphisms (SNPs) in the target sequence of the sgRNA (mismatch-based selectivity) or in the PAM (PAM-based selectivity). György and coworkers detected 1.3% indels in the APPsw allele after the hippocampal injection of a mismatch-based selective CRISPR/Cas9 system split into two AAV9 vectors (because of the limited capacity of AAV vectors of ∼4.8 kb) in Tg2576 mice (György et al., 2018). By contrast, Sun and coworkers used a non-allele selective CRISPR-mediated NHEJ strategy to push APP processing toward the non-amyloidogenic pathway (Sun et al., 2019). Based on evidence suggesting that deletion of the C-terminus of APP can mitigate Aβ generation (Koo and Squazzo, 1994) and reduce APP interactions with the BACE-1 enzyme (Das et al., 2016), the authors used CRISPR to generate C-terminally truncated APP, thereby circumventing the amyloidogenic processing of APP (Sun et al., 2019). In this study, APP truncation in WT and heterozygous APP-London human iPSC-derived neurons increased the production of the neuroprotective sAPPα and reduced the secretion of Aβ40/42 and the sAPPβ fragment. For *in vivo* studies in adult mice, the CRISPR-APP system was split into two AAV9 vectors and delivered to the dentate gyrus of WT mouse brains. The injection of CRISPR-APP reduces the level of the processed C-terminal fragments (CTFs) by half while having no or minimal impact on the total APP protein. No additional *in vivo* tests were performed to evaluate treatment efficacy in the context of AD (György et al., 2018; Sun et al., 2019), but these therapeutic strategies targeting the C-terminal part of APP are of interest because the aim was to attenuate pathological properties (Aβ generation) while potentially maintaining Duarte and Déglon Corrigendum: Genome Editing for CNS Disorders other physiological functions of APP. Another approach, developed by Park and coworkers, uses CRISPR-Cas9-loaded nanocomplexes targeting BACE1 in the 5XFAD and APP transgenic mouse models to reduce the generation of Aβ and improve AD symptoms (Park et al., 2019). Four weeks after CRISPR injection into the CA3 hippocampal region of 5XFAD mice, 45% of target sequences contained indels, and a 34% decrease in Bace1 expression was observed, revealing this method to be more efficient than the use of chemical BACE1 inhibitors. They also observed a decrease in Aβ plaque accumulation by a factor of more than two, together with a significant rescue of associative learning (fear conditioning test) and spatial working memory (Morris water maze) in the treated 5XFAD mice. These molecular and behavioral improvements were maintained for up to 12 weeks. Off-target evaluation by whole-genome sequencing (WGS), whole-exome sequencing (WES), Digenome-sequencing (Digenome-seq) and deep sequencing identified a few off-target mutations and small-scale chromosomal rearrangements.

Bustos and coworkers investigated the potential of epigenome editing for AD by targeting the dlg4 gene, encoding the PSD95 protein (Bustos et al., 2017). PSD95 is a scaffolding protein present at the excitatory post-synaptic density, and is involved in the regulation and organization of post-synaptic synapses (Elias and Nicoll, 2007). Abnormal PSD95 expression has been described not only in AD, but also in other neurological disorders, such as Huntington’s disease (HD) and schizophrenia. The authors developed and validated *in vitro* several ZF-based epigenome modifiers targeting the proximal promoter region of dlg4/PSD95 for the activation or repression of PSD95 expression (Bustos et al., 2017). They demonstrated that alterations in expression were specifically associated with histone modifications rather than other changes, such as CpG methylation in DNA. The fusion of zinc fingers to the histone methyltransferase G9a (PSD95-6ZF-G9a) induced gene repression associated with an increase in the di- and tri-methylation of H3K9, whereas PSD95-6ZF-VP64 gene activation was coupled to H3 activation, probably through the recruitment of histone acetylases by the VP64 domain. PSD95-6ZF-VP64 was also shown to have neuroprotective effects. AβPPswe/PS-1 mice receiving AAV-PSD95-6ZF-VP64 injections into the hippocampus had higher levels of PSD95 expression and displayed a rescue of memory and spatial learning performances to normal aged-matched levels.

### Parkinson’s Disease

Parkinson’s disease (PD) is the second most common neurodegenerative disorder, affecting 2–3% of people under the age of 65 years (Poewe et al., 2017). PD patients display motor movement dysfunction, but also cognitive impairment, depression and dementia. At the cellular and molecular levels, PD is characterized by a striatal dopamine deficiency due to progressive neuronal loss in the substantia nigra, and by the formation of intracellular aggregates containing α-synuclein. Dopamine loss and basal ganglia circuitry disruption are well-defined features in PD, but this disease is extremely complex and driven by diverse molecular and neurophysiological mechanisms.

Several gene-based therapies for PD have been proposed, including the targeting of α-synuclein, cellular oxidation and the autophagy-lysosomal pathway (Poewe et al., 2017). Genome editing for PD has mostly been used for disease modeling *in vitro* (Safari et al., 2019). For instance, Kantor and coworkers induced the hypermethylation of CpG islands in SNCA intron 1 in iPS-derived dopaminergic progenitor neurons, through lentiviral transduction with a dCas9-DNMT3A system (Kantor et al., 2018). They observed a ∼ 25% decrease in α-synuclein protein levels and the rescue of mitochondrial-associated superoxide production and cell viability. They observed no overall change in the methylation status of the treated cells, identifying the dCas9-DNMT3A-mediated targeting of SNCA as a promising approach for PD treatment. Another potential therapeutic target is glial cell line-derived neurotrophic factor (GDNF), which has been shown to have neuroprotective effects and to improve Parkinsonian symptoms (Kordower et al., 2000; Tenenbaum and Humbert-Claude, 2017). Laganiere and colleagues used a ZF-p65 fusion to upregulate the expression of endogenous GDNF in a 6-OHDA rat model of Parkinson’s disease (Laganiere et al., 2010). They observed an increase in the number of TH-positive fibers in both the medial forebrain bundle and the substantia nigra after 7 weeks of AAV2-rGDNF-ZFP infusion (Laganiere et al., 2010). The rGDNF-ZFP-treated group performed better in the corridor test, the cylinder test and the drug-induced rotational test than the GFP-treated control. This study yielded promising results, but a clinical trial based on the direct infusion of GDNF into the putamen resulted in no significant improvement of Parkinson’s disease symptoms (Lang et al., 2006; Whone et al., 2019), raising questions about therapeutic efficacy of GDNF.

### Huntington’s Disease

Huntington’s disease (HD) is a neurodegenerative disorder caused by an inherited dominant CAG trinucleotide expansion mutation on the HTT gene. *In vivo* genome editing strategies for HD have explored NHEJ-mediated gene inactivation (Merienne et al., 2017; Monteys et al., 2017; Yang et al., 2017) and the transcriptional repression of HTT (Zeitler et al., 2019). Yang et al. used two separate AAVs expressing SpCas9 and two sgRNAs targeting the flanking regions of the CAG repeat in a non–allele-specific manner in the HD140Q-KI mouse model (Yang et al., 2017). The injection of neuron-specific AAV-Cas9-HTT resulted in the efficient transduction of medium spiny neurons, significantly decreasing the accumulation of both mutant (mHTT) and WT HTT in the striatum of 9-month-old homozygous and heterozygous HD140Q-KI mice. The treated heterozygous mice performed better in the rotarod, beam and grip strength tests. Although no deleterious effects of depleting both mutant HTT copies from homozygous HD140Q-KI mice were detected (Yang et al., 2017), it still remains a matter of debate whether disruption of the normal physiological functions of WT HTT lead to harmful effects at adult stages (Liu and Zeitlin, 2017). With this in mind, Monteys and coworkers designed a PAM-based strategy targeting a SNP for specific inactivation of the mutant *HTT* allele (Monteys et al., 2017). They demonstrated the allele selectivity of the chosen sgRNAs *in vitro* in fibroblasts from human HD patients and showed efficient HTT exon-1-targeted deletion following the injection of allele-selective AAV1 CRISPR-HTT into BACHD transgenic mice. This treatment halved the levels of human mHTT mRNA in the striatum. However, it should be noted that heterozygous BACHD transgenic mice have about five tandem copies of the human mHTT gene and two copies of the endogenous mouse WT gene (Gray et al., 2008). In these studies, the spCas9 was constitutively expressed. The stable and permanent expression of nucleases eventually leads to higher levels of on-targeting editing, but it also increases the occurrence of off-target events and immunogenic responses. We have tried to overcome this problem by developing the self-inactivating KamiCas9 system, for transient Cas9 expression (Merienne et al., 2017). This system is based on a lentiviral vector with a larger cloning capacity than AAV. It is composed of the Cas9 nuclease, a sgRNA targeting HTT and a second sgRNA targeting the translation start site of the Cas9 nuclease. High on-target efficiency and inactivation of the Cas9 nuclease over time are ensured by the use of a strong PolIII promoter (H1) to drive the sgHTT and a weak PolIII promoter (7sk) to drive the sgCas9. We demonstrated high levels of exogenous hHTT-82Q (20–35%) and Cas9 (∼40%) editing following the injection of LV-KamiCas9 and hHTT-82Q into mouse striatum. Western blot analysis of striatal samples from mice receiving LV-KamiCas9 injections revealed an almost-complete absence of the Cas9 protein after 2 months.

Garriga-Canut et al. (2012) attempted to design a CAG copy number-dependent ZF-based transcription repressor exclusively targeting the mHTT allele. This first tool established the proof-of-principle for HTT repression *in vivo*, decreasing mHTT mRNA levels by about 30% in the brains of R2/6 mice receiving AAV1-ZF-Kox1 injections. Despite the achievement of selective repression *in vivo*, the mutant allele in R6/2 mice contains 115–160 repeats, a number not consistent with the degree of CAG expansion in most HD patients. Zeitler and coworkers recently generated a second-generation ZF-KRAB that preferentially recognizes pathogenic CAG repeats, and demonstrated highly significant mHTT suppression with wild-type allele preservation in patient derived-iPSCs (Zeitler et al., 2019). They observed beneficial behavioral effects in R6/2 mice for 7 weeks after the intrastriatal injection of AAV-ZF-KRAB, and demonstrated the absence of inflammation or adverse effects of long-term expression in mouse brain.

### Amyotrophic Lateral Sclerosis

Amyotrophic lateral sclerosis is a neurodegenerative disease caused by the progressive neurodegeneration of both upper and lower motor neurons (Rowland and Shneider, 2001). Muscle atrophy begins in adult patients with ALS and progresses to total paralysis and, eventually, death. Approximately 2% of ALS cases result from a dominant mutation of the SOD1 gene. Gaj et al. (2017) mitigated ALS symptoms and improved the survival of a mouse model of ALS, G93A-SOD1 mice, containing 25 copies of the human mutant SOD1, by disrupting the human SOD1 gene with the *Staphylococcus aureus* Cas9 (SaCas9). The CRISPR system was packaged into a single AAV9 variant (double-tyrosine mutant) shown to enhance gene transfer to the CNS (Petrs-Silva et al., 2009; Dalkara et al., 2012). The authors demonstrated efficient neuronal transduction of the ventral horn of the spinal cord, with up to 74% of motor neurons expressing the nuclease, after systemic injections in neonatal transgenic mice (Gaj et al., 2017). Western blot analysis revealed a 2.5- to 3-fold decrease in mutant SOD1 protein levels, but sequencing data showed that only a small fraction of the total human SOD1 transgenes had been edited (0.2–0.4%). This discrepancy may reflect the large numbers of glial cells in the gray matter of the spinal cord, which were not efficiently transduced, or differences in SOD1 expression in transduced and non-transduced regions of the spinal cord. Regardless of this divergence, the onset of disease in animals treated with SaCas9-SOD1 was delayed by 33 days, and survival was 28–30 days longer than in the control. In age-matched mice, the editing of SOD1 improved rotarod performance, prevented weight loss and reduced muscular atrophy. The treatment was unable to slow the progression of the disease after its onset, but end-stage tissue analysis in SaCas9-SOD1-treated mice revealed the presence of ∼50% more motor neurons. SOD1 inclusion bodies were observed in astrocytes, suggesting that glial cell targeting might be required to slow the progression of the disease, since these cells have been shown to play a role in disease progression (Boillée et al., 2006; Yamanaka et al., 2008).

### Angelman Syndrome

Angelman syndrome is a neurological disorder caused by a genetic UBE3A deficiency resulting in intellectual disability, ataxia and seizures (Laan et al., 1999). The paternal Ube3a allele is specifically silenced by a brain-specific antisense transcript (Ube3a-ATS). LOF mutations in the maternal allele therefore lead to UBE3A deficiency. Bailus and coworkers developed a ZF-KRAB repressor targeting the transcription start site of Ube3a-ATS (Snurf/Snrpn promoter), to overcome the paternal imprinting of the Ube3a gene (Bailus et al., 2016). The systemic injection of TAT-S1-linked UBE3a-6ZF-KRAB repressor partially rescued Ube3a expression levels in the hippocampus and cerebellum of a mouse model of Angelman syndrome. However, this therapeutic approach may require multiple treatments, because the repressor function of the KRAB domain has been shown to be transient (Gilbert et al., 2014; Ying et al., 2015).

### MECP2 Duplication Syndrome

MECP2 encodes a nuclear protein involved in the transcriptional and post-transcriptional regulation of many genes (Cheng and Qiu, 2014). Duplication or triplication of Xq28 leads to MECP2 GOF mutations mostly affecting boys (Ramocki et al., 2009). This syndrome is characterized by intellectual disability, poor speech development, motor dysfunction and anxiety. Yu and coworkers reported that the normalization of MeCP2 levels in the medial prefrontal cortex of adult MECP2 transgenic mice through CRISPR/Cas9-mediated NHEJ can reverse the social recognition deficit (Yu et al., 2020). The CRISPR system was packaged into two AAV particles (SpCas9 + sgRNA), which were stereotaxically injected into the mouse brain. Immunostaining and western blotting 6 weeks after treatment showed that MeCP2 protein levels had almost halved. Despite improvements in social recognition behavior, the treatment had no effect on locomotor activity, or heightened anxiety-like behaviors, suggesting that different brain areas or neural circuits may contribute to the diverse aspects of the syndrome.

### Fragile X Syndrome

Fragile X syndrome (FXS) is the most common single-gene form of autism spectrum disorders (ASDs), for which there is currently no effective treatment (Kaplan and McCracken, 2012). It is caused by a trinucleotide CGG repeat expansion in the 5′ UTR of the fragile X mental retardation 1 (FMR1) gene, encoding the fragile X mental retardation protein (FMRP) (Dölen and Bear, 2008; Persico and Napolioni, 2013). This mutation inactivates the gene, due to hypermethylation of the expanded repeats and heterochromatin formation. Excessive mGluR5 signaling has been observed not only in FXS, but also in other ASDs (Silverman et al., 2012). Lee and coworkers explored the CRISPR-mediated disruption of metabotropic glutamate receptor 5 (mGluR5) as a mean of counteracting FXS by delivering RNPs SpCas9 or Cas12a targeting the mGluR5 to the striatum of Fmr1-knockout mice (a mouse model of FXS) (Lee B. et al., 2018). The editing tool was delivered with CRISPR-gold technology, which combines gold nanoparticles conjugated with oligonucleotides and the endosomal disruptive polymer PAsp(DET), for the transfer of RNPs into cells by endocytosis (Lee et al., 2017). The indel frequency was 14.6%, and a 40–50% decrease in mGluR5 mRNA and protein levels was observed. In addition, behavioral analysis revealed that mGluR5-CRISPR-Gold rescued the excessive digging and exaggerated repetitive jumping behaviors of treated mice.

### Traumatic CNS Injury

Traumatic CNS injuries and stroke are very common causes of disability, and the treatments currently available are very limited. CNS trauma involves an initial mechanical injury, which is followed by a cascade of molecular and cellular phenomena, ultimately leading to neuronal death by apoptosis. Genome editing therapy strategies have focused on VEGF, which is a neuroprotective factor that favors endothelial cell proliferation and blood vessel formation (Shweiki et al., 1992). These studies used engineered ZFs targeting the proximal promoter of VEGF fused to the transactivating domain of the NF-kB p65 subunit. Michael Fehlings’s laboratory has demonstrated an increase in the number of blood vessels and angiogenesis, a decrease in neurodegeneration and an improvement of behavioral outcomes in a rat model of SCI following the intraspinal microinjection of AdV-ZFP-VEGF and AAV2-ZFP-VEGF activators (Liu et al., 2010). The timing of treatment for traumatic damage is an important parameter for clinical application. Beneficial effects have been shown following the administration of AdV-ZFP-VEGF 24 h after injury (Figley et al., 2014). In addition, Siddiq et al. (2012) used the unilateral fluid percussion injury model in rats to demonstrate the neuroprotective and angiogenic effects of ZFP-VEGF delivery to the cortex or hippocampus by intracerebral injection. Treatment did not improve performance in the Morris water maze or balance beam latency experiments relative to control, but the treated group performed significantly better than controls in the rotarod test.

### GM2-Gangliosidoses

GM2-gangliosidoses are autosomal recessive disorders caused by the deficiency of a lysosomal enzyme, β-hexosaminidase, resulting in the accumulation of GM2 gangliosides. Biallelic LOF mutations of the Hex α-subunit (HEXA) or Hex β-subunit (HEXB) genes lead to Tay-Sachs disease and Sandhoff disease, respectively. Ou and coworkers recently used a cross-correction strategy based on liver-targeted HDR-mediated CRISPR editing to restore the function of β-hexosaminidase in the brain, in a Sandhoff mouse model (Ou et al., 2020). They injected a dual AAV system consisting of AAV8-SaCas9 and AAV8-HEXM-sgRNA targeting the albumin safe harbor locus into neonatal Sandhoff mice, to introduce, via HDR, the coding sequence of a modified human Hex μ subunit (HEXM) able to process GM2 gangliosides (Karumuthil-Melethil et al., 2016). Four months after the systemic delivery of this sequence, levels of MUGS and MUG activity in the brain were significantly higher than those in untreated Sandhoff mice. Mice receiving the AAV8-HEXM-sgRNA alone displayed no such increase in MUGS and MUG activities, indicating an absence of HEXM expression from the episomal donor template vector. In addition, treated mice performed better in the rotarod test and one in three mice had lower levels of neuronal lysosomal accumulation, indicating that hepatocyte editing can lead to neurological improvements. Indeed, the HEXM variant has been reported to improve gangliosidosis in both the Sandhoff and Tay-Sachs models (Karumuthil-Melethil et al., 2016; Osmon et al., 2016), suggesting that this strategy may provide protection against both disorders.

### Hearing Loss Disorders (DFNA36 and DFNB7/11)

About 20% of the 100 or so alleles associated with deafness result from GOF mutations (Müller and Barr-Gillespie, 2015). DFNA36 is a progressive hearing loss disease caused by dominant mutations of the *tmc1* gene, leading to the neurodegeneration of sensory hair cells. This disease is of particular interest due to the existence of an orthologous mouse mutation, Beethoven (Bth), which also causes hearing loss in mice (Zhao et al., 2014). Two recent reports described the use *in vivo* of allele-specific CRISPR-mediated NHEJ as a therapeutic strategy for DFNA36 (Gao et al., 2018; György et al., 2019). Gao and coworkers used SpCas9 together with a sgRNA matching the mutant allele, but not the WT allele, to knockout the mutant allele (Gao et al., 2018). They delivered RNP complexes bound to cationic lipids and, even though the targeting of the mutant allele was highly selective (96% of mutant/WT), the frequency of indels was low (1.8%). Nevertheless, the treatment was sufficiently effective to promote hair cell survival, particularly for inner hair cells (IHCs), and to improve cochlear function significantly between the frequencies of 8 and 23 kHz (Gao et al., 2018). However, at 8 weeks, an analysis of cochlear function in treated Tmc1Bth/ + mice revealed less evident improvements relative to the control, suggesting that higher levels of mutant gene inactivation might be required to stop neurodegeneration, or that the small proportion of WT alleles inactivated might neutralize the benefits of mutant knockout over time. This strategy resulted in allele-specific editing, but PAM-based strategies are generally more selective, as demonstrated by György and coworkers (György et al., 2019). They used the SaCas9-KKH variant to edit the mutant allele in a PAM selective manner (György et al., 2019). The SaCas9-KKH/sgRNA treatment via AAV-Anc80L65 was more selective that the treatment used in the previous study, with no detectable indels in the WT allele and a frequency of 2.2% indels for the mutant allele. At the age of 6 months, SaCas9-KKH/sgRNA-treated mice had significantly higher survival rates for both inner hairy cells and outer hair cells (OHCs), with normal hair bundle morphology in all cochlea, except for the OHCs in the basal region, which were absent. The authors also demonstrated the stable maintenance of low thresholds of auditory brainstem responses for up to 40 weeks. Finally, GUIDESeq analysis detected no genome-wide off-target events in Tmc1WT/WT fibroblasts, further highlighting the potential interest of AAV-SaCas9-KKH-sgTmc as a therapeutic strategy for DFNA36 hearing loss.

DNFA36 results from GOF mutations of the *tmc1* gene, whereas LOF mutations in both *tmc1* alleles result in the autosomal recessive congenital DFNB7/B11 hearing loss disorder. Gene disruption approaches are not suitable for the treatment of DFNB7/B11. Yeh and coworkers explored the use of a base editing strategy to correct the *tmc1* alleles in *Tmc*1^Y182C/Y182C^ mice (Yeh et al., 2020). They reported 2.3% base editing in a bulk organ of Corti at P14 after the injection of a dual AAV system encoding AID-BE into the inner ear at P1. Tmc1 is expressed only in hair cells. The authors therefore analyzed base editing at the RNA level, to improve the quantification of editing in these cells. They observed ∼ 50% editing in the cDNA, but these results must be interpreted with caution because they may not reflect the editing at the DNA level. The treatment of mice resulted in the preservation of hair bundle morphology and a restoration of the mechanotransduction current in the sensory hair cells. There were 46% more hair cells in the treated mice 4 weeks after injection, with a progressive decrease in cell numbers thereafter, until 6 weeks. The decrease in cell survival was followed by a decline in hearing function, suggesting that more efficient base editing is required to prevent the degeneration of hair cells over time.

### Retinitis Pigmentosa

Retinitis pigmentosa (RP) is an inherited disorder and the most common cause of progressive vision loss (Kalloniatis and Fletcher, 2004). It is defined by an initial progressive loss of rod photoreceptors, followed by cone photoreceptor degeneration. One form of RP results from a biallelic LOF mutation in the PDE6B gene, introducing a premature stop codon. Cai et al. used HDR-mediated CRISPR editing to correct the mutation (Cai et al., 2019). In this study, the authors developed an improved CRISPR system for HDR (Cas9/RecA) consisting of a sgRNA with MS2 aptamers for the recruitment of MS2-RecA fusion proteins to the target site to promote recombination between the cleavage site and a ssDNA donor template. The potential of this tool to repair the PDE6B gene was evaluated by electroporating the retinas of WT and rd1 mice with four plasmids (SpCas9 + sgRNA-MS2apt + MS2-RecA + ssTemplate) at P0. A 2% restoration of PDE6B WT protein levels was observed in Cas9/RecA-treated mice, whereas no wild-type PDE6B protein was detected in Cas9-treated mice (SpCas9 + sgRNA-MS2apt + ssTemplate), indicating that Cas9/RecA enhances HDR efficiency. Cas/RecA treatment at P0 rescued both rod and cone photoreceptors, but the degree of rescue was 1.8- and 1.6-fold lower, respectively, when mice were treated at P3, suggesting that the loss of photoreceptor proliferation had a negative effect on HDR-mediated correction. In addition, an analysis of visual function and pupillary light reflexes revealed that Cas9/RecA partially rescued the pupillary light reflexes of rd1 mice, demonstrating beneficial effects of treatment.

### Leber Congenital Amaurosis Type 10

Leber congenital amaurosis type 10 (LCA10) is an autosomal recessive condition causing early blindness in infancy (Stone, 2007; Stone et al., 2017). It is defined by LOF mutations of both *CEP290* alleles. The IVS26 point mutation creating a new splice donor site is the most frequent defect. It alters transcript splicing and generates a premature stop codon in the processed mRNA. Maeder and coworkers recently reported an exhaustive drug dosing study of the use of AAV5-SaCas9-mediated NHEJ to correct the IVS26-driven aberrant CEP290 splicing in retina photoreceptor cells (EDIT-101) (Maeder et al., 2019). The proposed strategy induced a cleavage on either side of the mutation, with a pair of sgRNAs used to delete or invert the fragment containing the IVS26 mutation. The authors evaluated the kinetics and dose response of the editing system in the retina of CEP290 IVS26-KI mice and cynomolgus monkeys, in which maximum editing rates of 21.4 and 27.9%, respectively, were obtained. They also demonstrated ocular tolerability in all animals, except those without immunosuppression regimens, which displayed mild inflammation. This report resulted in the first approved preclinical study of CNS genome editing for clinical trial continuation in humans (NCT03872479). The *cep290* cDNA is ∼7.5 kb long, a size well-beyond the capacity of the AAV vectors used for gene replacement. This approach demonstrates the therapeutic potential of gene repair for counteracting CNS disorders without the need to provide exogenous WT transgenes.

## Future Perspectives in Genome Editing for CNS Disorders

The field of genome editing is rapidly evolving and there is now a broad genome editing toolbox that can be used for therapeutic purposes. The efficacy of genome-editing therapies for CNS disorders will depend on the choice of the most appropriate tool to tackle the genetic defect and the type and magnitude of editing required for therapeutic benefit. In addition, the types of cells and CNS areas to be edited should be taken into account. Local genome editing may be sufficient for some disorders, but others may require the editing of large areas. For instance, eye disorders are more accessible due to their peripheral localization and the relatively small area targeted, whereas the neuronal damage in AD covers large brain regions (Dos Santos Picanco et al., 2018). It is, therefore, crucial to select the most suitable delivery vehicle according to the editing tool used and the target area. The delivery of genome editing tools is probably one of the major limiting steps when targeting the CNS. Viral-mediated delivery by lentiviral (LV) and AAV vectors is the approach most frequently used to date, due to their high efficiency to transfer genetic material into cells (Spencer et al., 2020). LV have a large loading capacity but integrate into the host genome, potentially leading to insertional mutagenesis, whereas AAVs mostly persist as an extrachromosomal episome but have a limited cloning capacity. The generation of non-integrative lentiviral vectors (Shaw et al., 2017) and the use of dual AAV delivery systems (Yang et al., 2017; György et al., 2018; Sun et al., 2019) are two alternatives for overcoming these problems. Viral tropism has also been used to target specific cell types and to increase the area of transduction by either viral neuronal retrograde transport or through the use of serotypes with wide diffusion properties (Lykken et al., 2018). Local intraparenchymal injections are the most common delivery method for circumventing the BBB, but some AAV serotypes have been shown to cross the BBB after systemic delivery (Choudhury et al., 2016b; Chan et al., 2017; Hudry et al., 2018). Non-viral vehicles are generally less efficient than viral vectors, but the development of non-viral delivery methods for the CNS is an intense field of research and may open up new possibilities for treatment in the near future (Wang and Huang, 2019).

The immunogenicity induced by genome editing tools is another topic of concern due to potential inflammatory responses (Shim et al., 2017). For instance, the injection of non-host-matched, but not host-matched ZFNs, into the mouse brain resulted in microglial activation and mild neuronal death (Agustín-Pavón et al., 2016). Similarly, CRISPR/Cas9 was shown to induce the both cellular and humoral immune responses in mouse models (Mehta and Merkel, 2020). Immunogenicity can be minimized by transient expression. Transient expression strategies have been mainly developed for CRISPR/Cas-based tools either through the delivery of RNPs or ON/OFF expression systems. These include self-inactivation systems (Merienne et al., 2017; Li et al., 2018) and the use of drug inducible promoters, such as the doxycycline (dox)-induced Tet or the Tamoxifen-dependent Cre promoters (Zhang et al., 2019). However, the optimization of self-inactivation kinetics and the requirement of additional molecules to regulate promoters will delay the translation of these strategies to the clinic. Additionally, these strategies will be only suitable if the transient expression of the tool is sufficient to achieve therapeutic benefit.

Transient systems have also been developed to decrease off-target modifications. We showed that off-target events were reduced with the KamiCas9 compared to the constitutively expressed Cas9 (Merienne et al., 2017). Other groups have engineered Cas9 binding properties to increase specificity and attenuate off-target editing (Cebrian-Serrano and Davies, 2017; Hu et al., 2018; Kocak et al., 2019). Similarly, base editors and transcriptional/epigenetic editors also present off-target effects. For instance, base editors induce off-targets at both DNA and RNA levels (Zhou et al., 2019) whereas the KRAB domain has been shown to affect long chromosomal regions (Groner et al., 2010). There is thus the need for the development of highly specific editing systems to minimize safety concerns and ease their clinical application.

Finally, on-target events should also be properly characterized. Gene editing generates chimeric outcomes by introducing heterogeneous indels. For instance, the CRISPR/Cas9 targeting of the HTT translation starting site followed by NHEJ may generate truncated proteins with polyserine or polyalanine expansions, which have been shown to play a role in the disease (Berger et al., 2006). Furthermore, when attempting HDR-based strategies, NHEJ and HDR are competing pathways, and DSBs may be repaired by both mechanisms in the presence of a repair template (Weisheit et al., 2020). Likewise, bystander editing during base editing may give rise to unintended edited products which might even intensify the pathological processes. In addition, genome editing events may also be neutralized by intrinsic compensatory mechanisms, reducing the therapeutic effects (Smits et al., 2019).

In this review, we have focused on examples of *in vivo* therapies for CNS disorders (Table 2), but extensive efforts have been conducted to improve genome editing strategies. Two examples are the recently proposed prime editing approach (Anzalone et al., 2019) and the usage of transposases for genome engineering, which may become alternative options for the treatment of CNS disorders in the near future (Anzalone et al., 2020; Doudna, 2020). In summary, it is acknowledged that multiple aspects require further improvement to establish CNS genome-editing therapies but the field is advancing at an astonishing pace, bringing us closer every day to possible clinical applications.

**TABLE 2:** Preclinical studies of genome editing for CNS pathologies.

Editing approach	Disease	Gene	Model	Editing tool	Delivery	Selectivity	Target efficiency (indels/expression)	Behavioral improvements	Publication
**NHEJ**	MECP2 duplication syndrome	Mecp2	MECP2-TG mouse	SpCas9	AAV-split system	Non-selective	50% reduction MECP2 protein	Improvements in social recognition	Yu et al., 2020
	Fragile X syndrome	mGluR5	Fmr1 knockout mouse	SpCas9	CRISPR-Gold RNP complexes	Non-selective	14.6% indels 40–50% reduction mGluR5 mRNA and protein	Rescued the excessive digging and repetitive jumping	Lee B. et al., 2018
	Alzheimer’s disease	Bace1	5XFAD and APP-KI mouse	SpCas9	Amphiphilic RNP complexes	Non-selective	45% indels 34% reduction Bace1 mRNA	Behavioral improvements	Park et al., 2019
		APP	WT mouse	SpCas9	AAV9-split system	Non-selective	50% reduction full-lenght APP protein	No data	Sun et al., 2019
		APP-SW	Tg2576 mouse	SpCas9	AAV9-split system	Mismatch-based	1.3% indels (APPsw alleles)	No data	György et al., 2018
	DFNA36 (hearing loss)	Tmc1	Beethoven mouse (Bth/wt)	SpCas9	Cationic lipid-mediated RNP complexes	Mismatch-based	1.8% indels (mutant alleles)	Protection of the acoustic behavioral reflexes	Gao et al., 2018
		Tmc1	Beethoven mouse (Bth/wt)	SaCas9-KKH	AAV-Anc80L65	PAM-based	2.2% indels (mutant alleles)	Stable maintenance of auditory brainstem responses	György et al., 2019
	ALS	SOD1	G93A-SOD1 mouse	SaCas9	AAV9	Non-selective	0.2–0.4% indels 65% reduction SOD1 protein	Improved survival, motor deficits and muscular strenght	Gaj et al., 2017
	Huntington’s disease	HTT	HD140Q-KI mouse	SpCas9	AAV-split system	Non-selective	10–80% reduction HTT protein	Improved motor deficits	Yang et al., 2017
		HTT	BacHD mouse	SpCas9	AAV1-split system	PAM-based	50% reduction mHTT mRNA	No data	Monteys et al., 2017
		HTT	LV-hHTT-82Q mouse	SpCas9 (self-inactivating)	LV-split system	Non-selective	30% HTT indels (exogenous)	No data	Merienne et al., 2017
	LCA10	CEP290	CEP290 IVS26-KI mouse and monkeys	SaCas9	AAV5	Non-selective	21.4% and 27.9% indels	No data	Maeder et al., 2019
**HDR**	Sandhoff and Tay–Sachs diseases	ALB	Sandhoff mouse	SaCas9 + dsTemplate-HEXM	AAV8	Non-selective	144- and 17-fold increase MUGS and MUG activities (indirect)	Improved motor deficits (totarod test)	Ou et al., 2020
	Retinitis pigmentosa	Pde6b	Rodless (rd1) mouse	SpCas9 + RecA-MS2 + sgRNA-MS2 loops + ssTemplate	Plasmid electroporation	Non-selective	2% gene correction	Partial rescue of the pupillary light reflexes	Cai et al., 2019
**Base editing**	DFNB7/B11 (hearing loss)	Tmc1	Tmc1 (Y182C/Y182C) mouse	SpCas9-based AID-BE4max	AAV-Anc80L65-split system	Non-selective	2.3% gene correction	Improved auditory brainstem responses	Yeh et al., 2020
**Transcriptional activation**	Parkinson’s disease	GDNF	6-OHDA rat	GDNF-6ZF-p65	AAV2	Non-selective	60% increase GDNF mRNA	Rescued motor deficits	Laganiere et al., 2010
	Alzheimer’s disease	Dlg4	AβPPswe/PS-1 mouse	PSD95-6ZF-VP64	AAV-PHP.B	Non-selective	31% increase Bace1 mRNA	Rescued memory deficits	Bustos et al., 2017
	Spinal cord injury	VEGF-A	Aneurysm clip compression rat	VEGF-3ZF-p65	Ad and AAV2	Non-selective	33% increase VEGF mRNA 55% increase VEGF protein	Improved motor deficits	Liu et al., 2010;
	Traumatic brain injury	VEGF-A	Unilateral FPI rat	VEGF-3ZF-p65	Ad and AAV2	Non-selective	25–50% increase VEGF protein	Improved motor deficits (rotarod test)	Siddiq et al., 2012
**Transcriptional repression**	Huntington’s disease	HTT	R6/2 mouse	mHTT-6ZF-KRAB	AAV1	CAG selective (120 repeats)	30% reduction mHTT mRNA	Improved motor deficits and clasping behavior	Garriga-Canut et al., 2012
		HTT	R6/2 and HdhQ50 mouse	mHTT-6ZF-KRAB	AAV1	CAG selective (50 repeats)	55–67% reduction mHTT mRNA	Improved clasping behavior	Zeitler et al., 2019
	Angelman syndrome	Snurf/Snrpn	Maternally Ube3a-deficient mouse	UBE3a-6ZF-KRAB	HIV TAT cell-penetrating peptide	Non-selective	20% increase UBE3A mRNA (indirect)	No data	Bailus et al., 2016

## Author Contributions

Both authors listed have made a substantial, direct and intellectual contribution to the work, and approved it for publication.

## Conflict of Interest

The authors declare that the research was conducted in the absence of any commercial or financial relationships that could be construed as a potential conflict of interest.
