# Corrigendum: Genome Editing for CNS Disorders

**DOI:** 10.3389/fnins.2021.698879

**Published:** 2021-05-26

**Authors:** Fábio Duarte, Nicole Déglon

**Affiliations:** ^1^Laboratory of Neurotherapies and NeuroModulation, Department of Clinical Neurosciences, Lausanne University Hospital and Lausanne University, Lausanne, Switzerland; ^2^Laboratory of Neurotherapies and NeuroModulation, Neuroscience Research Center, Lausanne University Hospital and Lausanne University, Lausanne, Switzerland

**Keywords:** CNS, genome editing, ZFs, TALEs, CRISPR/Cas

In the original article, we inaccurately cited the report of Sun et al. (2019). The authors did not show a 50% decrease of the total APP protein as indicated in our review. Instead, they showed that APP editing reduces the level of the processed C-terminal fragments (CTFs) by half while having no or minimal impact on the total APP protein.

A correction has been made to *Genome Editing for CNS Disorders, Alzheimer's Disease, Paragraph 1*. The corrected paragraph is shown below.

Alzheimer's disease (AD) is the main cause of dementia, affecting millions of people worldwide (Winblad et al., 2016; Dos Santos Picanco et al., 2018). One of the hallmarks of AD is the presence of scattered extracellular senile plaques, due to the accumulation of amyloid-β (Aβ) in the brain. Aβ is a secondary metabolite generated by the processing of amyloid precursor protein (APP) by β-secretase 1 (BACE1). Alternatively, APP may be processed via a non-amyloidogenic pathway involving α-secretases, leading to the generation of neuroprotective products (Richter et al., 2018). In a study of the treatment of a familial form of AD caused by the Swedish mutation of APP (APPsw), CRISPR-mediated NHEJ was used to inactivate the mutant APP (György et al., 2018). This can be achieved by designing sgRNAs targeting single nucleotide polymorphisms (SNPs) in the target sequence of the sgRNA (mismatch-based selectivity) or in the PAM (PAM-based selectivity). György and coworkers detected 1.3% indels in the APPsw allele after the hippocampal injection of a mismatch-based selective CRISPR/Cas9 system split into two AAV9 vectors (because of the limited capacity of AAV vectors of ≈4.8 kb) in Tg2576 mice (György et al., 2018). By contrast, Sun and coworkers used a non-allele selective CRISPR-mediated NHEJ strategy to push APP processing toward the non-amyloidogenic pathway (Sun et al., 2019). Based on evidence suggesting that deletion of the C-terminus of APP can mitigate Aβ generation (Koo and Squazzo, 1994) and reduce APP interactions with the BACE-1 enzyme (Das et al., 2016), the authors used CRISPR to generate C-terminally truncated APP, thereby circumventing the amyloidogenic processing of APP (Sun et al., 2019). In this study, APP truncation in WT and heterozygous APP-London human iPSC-derived neurons increased the production of the neuroprotective sAPPα and reduced the secretion of Aβ40/42 and the sAPPβ fragment. For *in vivo* studies in adult mice, the CRISPR-APP system was split into two AAV9 vectors and delivered to the dentate gyrus of WT mouse brains. The injection of CRISPR-APP reduces the level of the processed C-terminal fragments (CTFs) by half while having no or minimal impact on the total APP protein. No additional *in vivo* tests were performed to evaluate treatment efficacy in the context of AD (György et al., 2018; Sun et al., 2019), but these therapeutic strategies targeting the C-terminal part of APP are of interest because the aim was to attenuate pathological properties (Aβ generation) while potentially maintaining other physiological functions of APP. Another approach, developed by Park and coworkers, uses CRISPR-Cas9-loaded nanocomplexes targeting BACE1 in the 5XFAD and APP transgenic mouse models to reduce the generation of Aβ and improve AD symptoms (Park et al., 2019). Four weeks after CRISPR injection into the CA3 hippocampal region of 5XFAD mice, 45% of target sequences contained indels, and a 34% decrease in BACE1 expression was observed, revealing this method to be more efficient than the use of chemical BACE1 inhibitors. They also observed a decrease in Aβ plaque accumulation by a factor of more than two, together with a significant rescue of associative learning (fear conditioning test) and spatial working memory (Morris water maze) in the treated 5XFAD mice. These molecular and behavioral improvements were maintained for up to 12 weeks. Off-target evaluation by whole-genome sequencing (WGS), whole-exome sequencing (WES), Digenome-sequencing (Digenome-seq) and deep sequencing identified a few off-target mutations and small-scale chromosomal rearrangements.

The authors apologize for this error and state that this does not change the scientific conclusions of the article in any way. The original article has been updated.
